# Supplementary material for: Trajectory-modulated hippocampal neurons persist throughout memory-guided navigation
Source: Nat Commun. 2020 May 15;11:2443. doi: 10.1038/s41467-020-16226-4 (PMC7229120; doi:10.1038/s41467-020-16226-4)
Supplement: Supplementary file 1 — Supplementary Information [file 41467_2020_16226_MOESM1_ESM.pdf]

Supplementary Figure 1

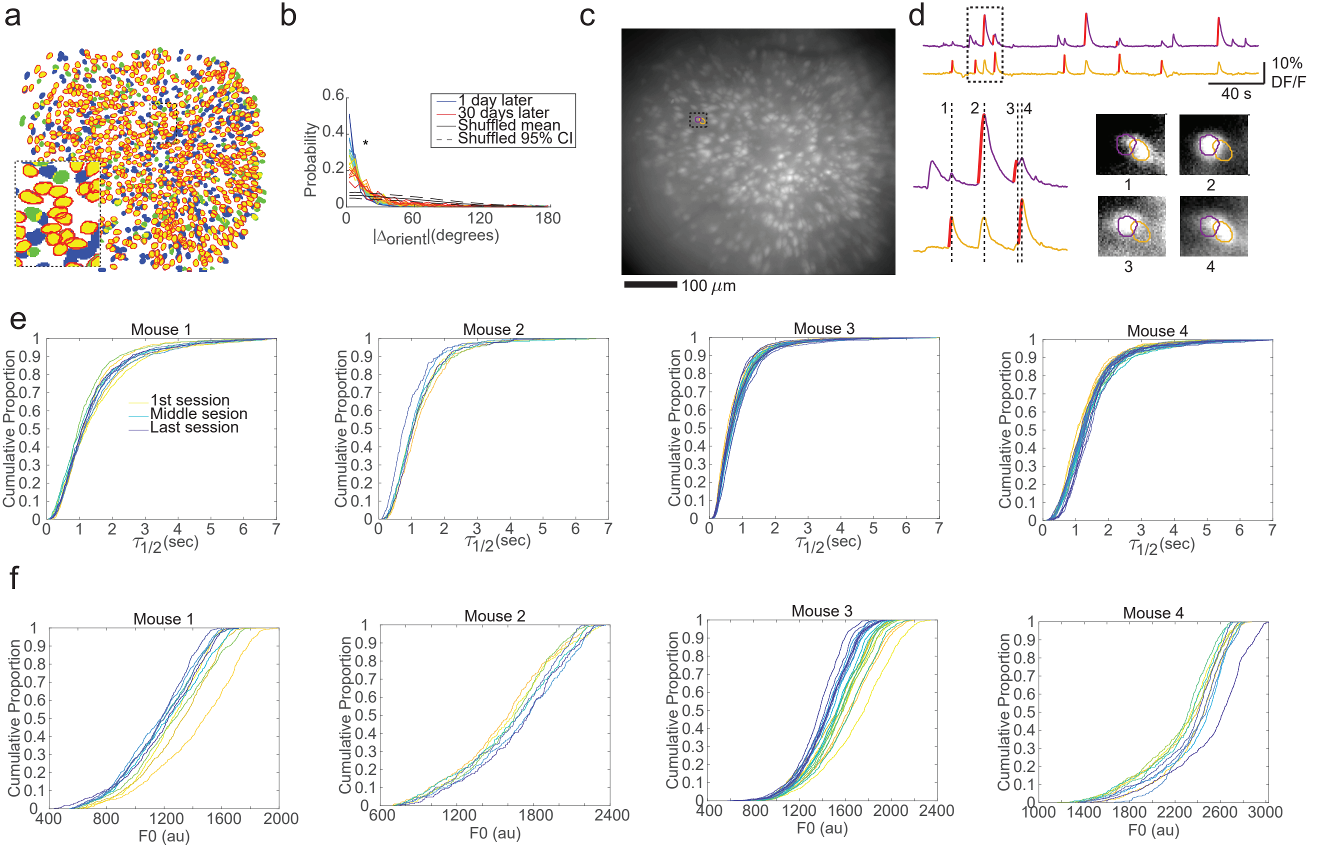

Supplementary Figure 1 | Neuron Extraction, Across-Session Registration, and Imaging Properties Across Days. Related to Figure 1.

a Example neuron registration between two sessions. Blue/Green = pixels corresponding to putative ROIs extracted in the 1st/2nd session only. Yellow = pixels corresponding to portions of ROIs active in both sessions. Red = outline of ROIs matched as the same neuron between sessions. **b** Minimal changes in ROI orientation between sessions indicate proper neuron registration between sessions. *p<9.04x10-6 (n = 152-831 neurons) for all session-pairs, one-sided Kolmogorov-Smirnov test versus distribution obtained after shuffling cell map between session (1000 shuffles). Significance assessed at α = 0.05 after Bonferroni correction (25 session-pairs). **c** Maximum projection of 1st imaging session shown in a (same projection as shown in Figure 1d). Dashed box shows two neurons with overlapping ROIs. See Figure 1d for statement on the reproducibility of imaging and number of neurons detected per session. **d** (top) Calcium traces for overlapping neuron ROIs in c. Red = putative spiking epoch for that neuron ROI. (bottom left) Expansion of calcium traces shown in c demonstrating assignment of calcium events to the appropriate ROI. Dashed line = peak fluorescence of each calcium event. Note that each event for one ROI produces a smaller increase in fluorescence in the neighboring ROI. (bottom right) Close-up of imaging movie frames for times in bottom left demonstrates fluorescence centered in ROI to which the event corresponds in bottom left. **e** Cumulative density plot of half-decay times for neurons across all recording sessions indicates highly similar and stable calcium kinetics across the entire experiment. **f** Cumulative density plot of mean fluorescence values for all neurons across all recording sessions shows no sign of increased fluorescence across sessions. Note that Mouse 4 was imaged using two different excitation powers and only data from the higher power is shown here for a fair comparison.

Supplementary Figure 2

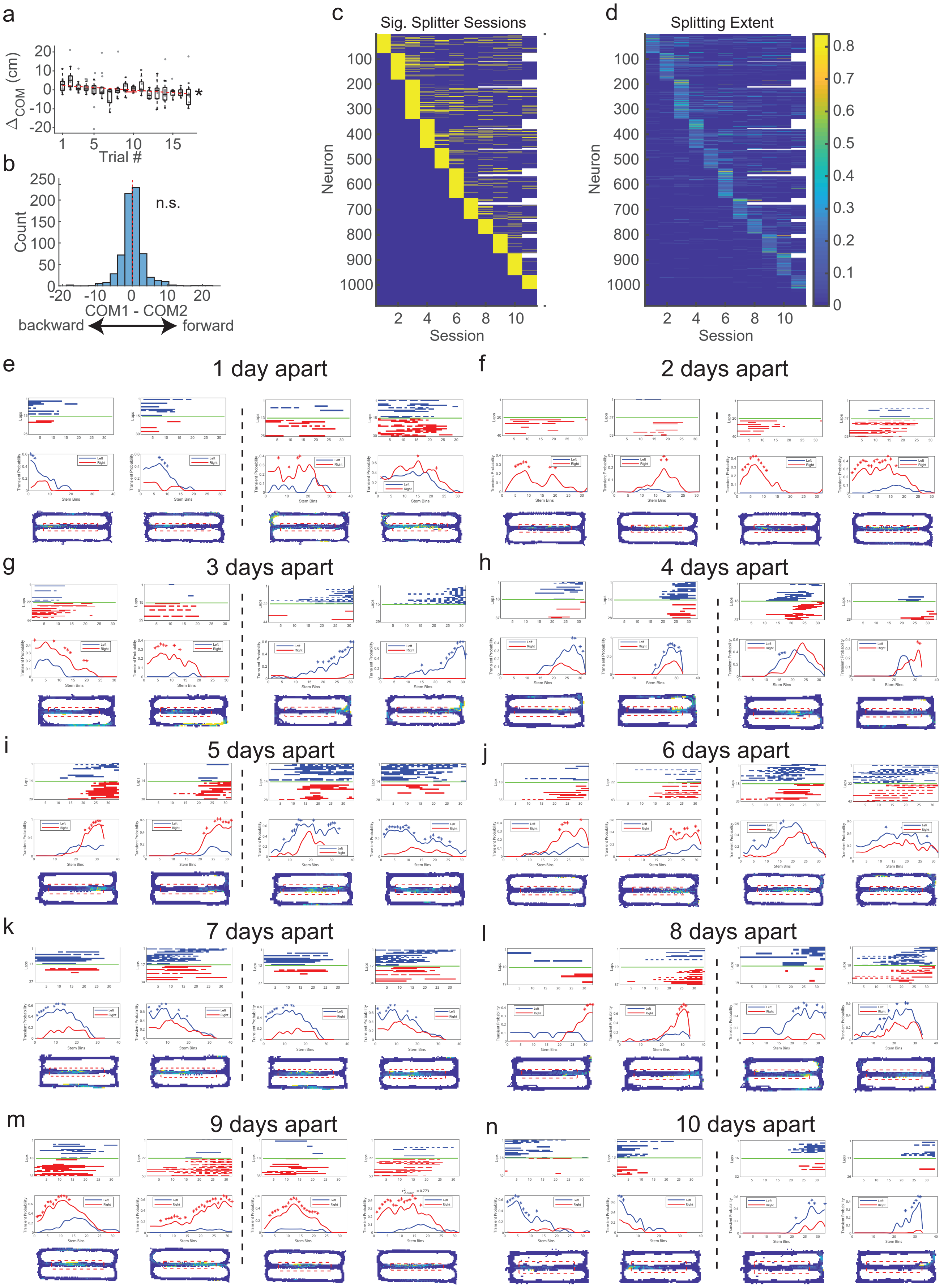

Supplementary Figure 2 | Trajectory-Dependent Activity Persists Across Multiple Sessions while Spatial Activity Migrates Backwards within but not between Sessions. Related to Figure 2.

a The centroid of spatial firing of all splitter neurons on the stem relative to their mean locations across the entire session drifts backwards throughout the session (left trials only shown). *$r = -0.28$, $p=5.1e-5$, $t = -4.1$ for null hypothesis that slope = 0, two-sided t-test ($\alpha=0.05$, significant after Bonferroni correction for $n = 68$ sessions), $n = 175$ centroid-shifts. Circles = centroid shifts for each neuron active on the stem. Example session from one mouse for right turns only. Box plots show median and 1st/3rd quartiles, whiskers extend to 1.5x interquartile range. **b** Average change in spatial firing centroid location between adjacent sessions for all mice between two sessions indicates that place field location does not drift between sessions. $p = 0.67$, two-sided t-test, $n = 685$ session-pairs. **c** Summary plot of all splitter neurons tracking if they exhibited significant trajectory-dependent activity (yellow) on up to 10 subsequent recording sessions (blue = no activity or non-significant trajectory-dependent activity, white = after last recording session for that mouse). Note that since imaging planes shifted approximately halfway through the experiment for mouse 3 and mouse 4, we treated the second half of recordings for each mouse independently in order to accurately track the longevity of all neurons we recorded. **d** Same as c but for splitting extent (the proportion of stem bins exhibiting significant trajectory-dependent activity). **e–n** Example neurons displaying trajectory-dependent activity across sessions 1-10 days apart, conventions as shown in Figure 1a-b with 2D event rate maps rotated 90 degrees counterclockwise. Each subpanel contains two neurons different neurons (separated by the dashed line) separated between sessions from one mouse, with all four mice represented in at least one subpanel. Note backwards migration is readily apparent in almost all neurons, in particular g, h (left), j (right), and l (right). Same conventions as Figure 2a-b.

Supplementary Figure 3

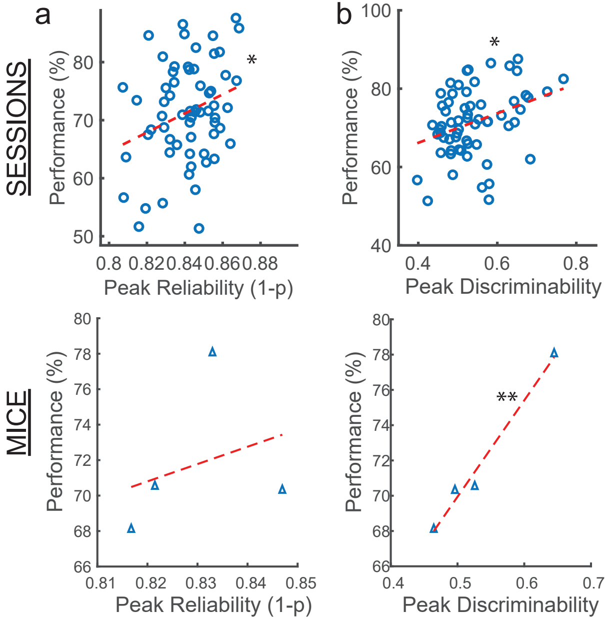

Supplementary Figure 3 | The Quality of Local Trajectory-Dependent Information Correlates with Performance. Related to Figure 3.

a (top) Performance for each session versus the peak discriminability value for all cells from that session. Circles = all sessions, all mice. (bottom) Same as (top) but for each mouse, triangles = average for each mouse. *ρ=0.29, p=0.023 Pearson correlation.
b Same as a, but for the peak reliability value. *ρ=0.034, p=0.0062, **ρ=0.98, p=0.0094 Pearson correlation.

Supplementary Figure 4

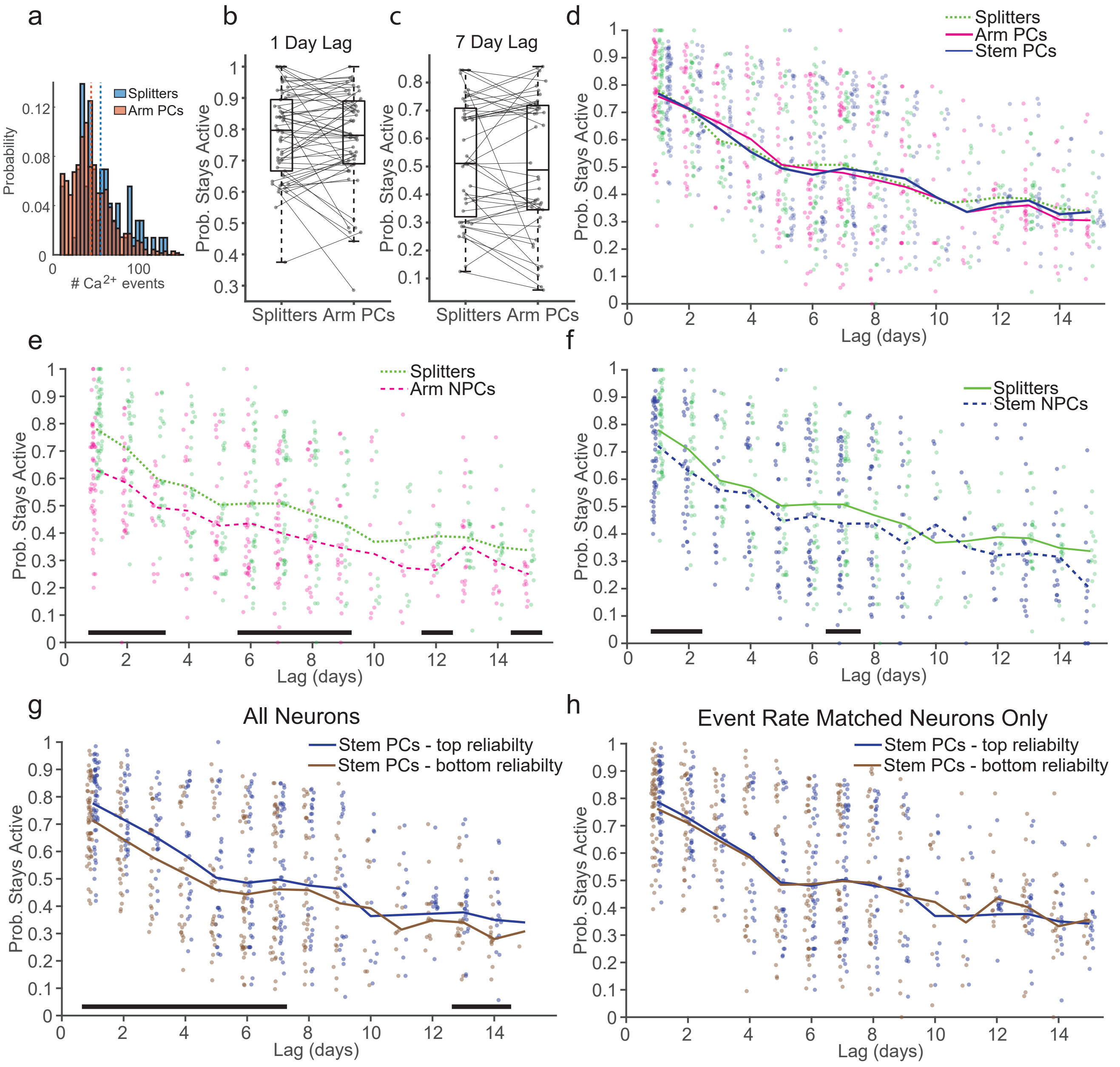

Supplementary Figure 4 | Turnover Rates for Splitter Neurons versus Event-Rate Matched Place Cells and Non-Place Cells. Related to Figure 4.

a Histogram from one session showing that Ca2+ event rates are higher for splitters for than for arm PCs. Blue/red dashed: mean number Ca2+ events for splitter/arm PCs. **b** Probability splitters and a subset of PCs (subsampled such that the mean event rate matches that of splitter neurons for each session) stay active one day later. p=0.56, one-sided signed-rank test, n = 58 session-pairs. Box plots show median and 1st/3rd quartiles, whiskers extend to 1.5x interquartile range. **c** Probability splitters and PCs stay active seven days later for all mice. p=0.31, one-sided signed-rank test, n = 44 session-pairs. Same conventions as b. **d** Probability splitters and arm place cells stay active versus lag between sessions. Dots: probabilities from individual session-pairs, lines: mean probability at each time lag. Green/magenta/blue: splitters/arm PCs/stem PCs. Black bars = significant differences after Holm-Bonferroni correction (15 day lags considered) of one-sided sign test, α = 0.05. See Table 3 for one-sided signed-rank test p-values at all lags.**e** Same as d but for splitters (red) vs. arm NPCs (red dashed). **f** Same as d for splitters vs. stem NPCs. **g** Same as d but for stem PCs with the most reliable (top quartile reliability score) vs. least reliable (bottom quartile) trajectory-dependent activity including all neurons. See Table 2 for one-sided signed-rank test p-values at all lags. **h** Same as g but using a subset of neurons such that each group has the same mean event rate.

Supplementary Figure 5

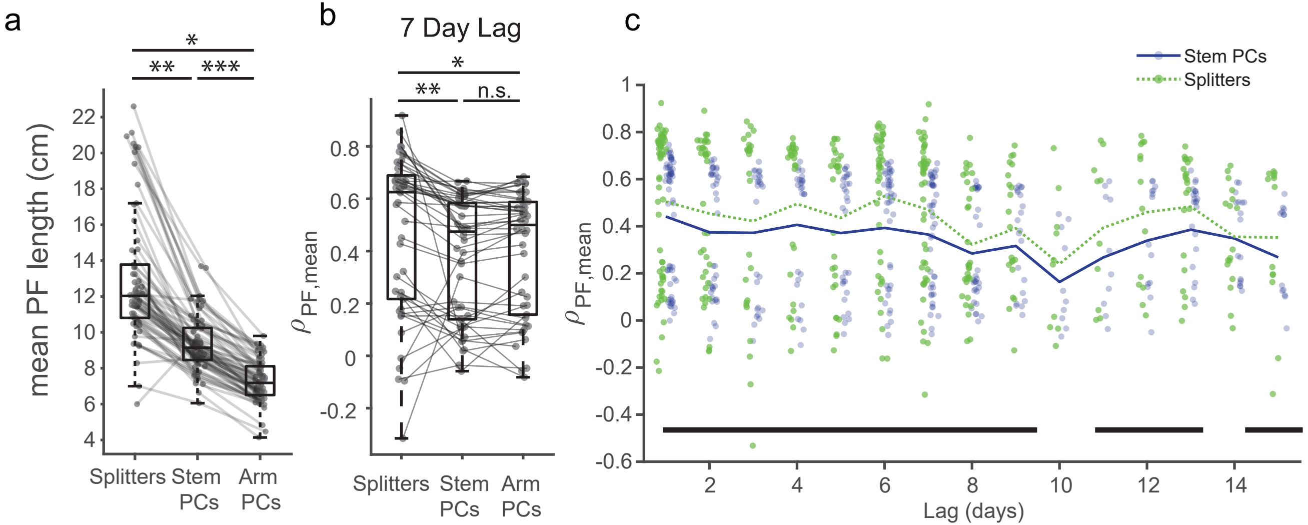

Supplementary Figure 5 | Spatial Consistency of Splitters vs. Stem and Arm Place Cells (PCs). Related to Figure 5.

a Mean place-field length for splitter neurons, stem PCs, and arm PCs across all sessions. *p=1.7x10-12, **p=1.2x10-11, ***p=7.6x10-13 one-sided signed-rank test, n = 68 sessions. Box plots show median and 1st/3rd quartiles, whiskers extend to 1.5x interquartile range. **b** Mean spatial correlations for splitter neurons versus stem PCs and arm PCs for all sessions seven days apart from all mice. *p=1.7x10-5, **p=1.1x10-5, n = 46 session-pairs, one-sided signed-rank test. Same conventions as a. **c** Mean spatial correlations for splitter neurons and stem PCs versus lag between sessions for all mice/sessions. Blue = Stem PCs, green dashed = splitters, black bars = significant differences after Holm-Bonferroni correction of one-sided sign-test, α = 0.05. See Table 4 for p-values at all lags.

Supplementary Figure 6

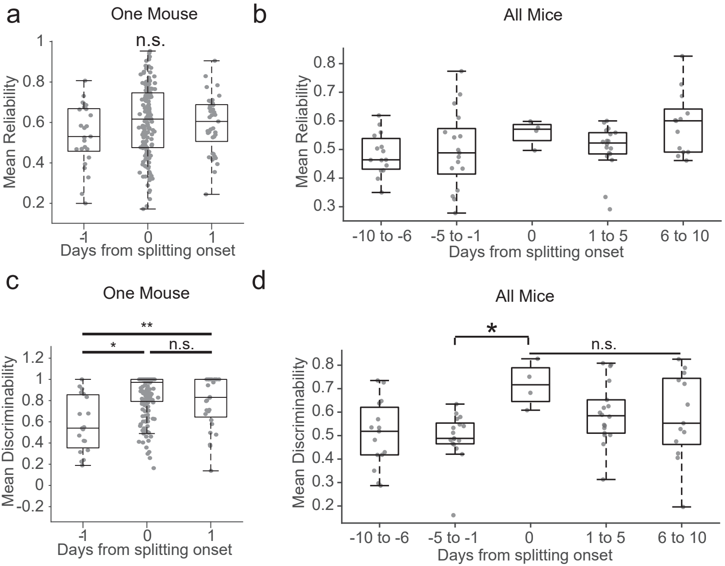

Supplementary Figure 6 | Tracking the Onset of Trajectory-Dependent Activity along the Entire Stem. Related to Figure 6.

a Mean reliability along the stem +/- 1 days from splitter onset for one representative mouse. p=0.15 Kruskal-Wallis ANOVA, N = 221 neurons. Box plots show median and 1st/3rd quartiles, whiskers extend to 1.5x interquartile range. **b** Mean reliability +/- 10 days from splitter onset for all mice. p=0.049 Kruskal-Wallis ANOVA, N = 67 mean reliability scores. Circles = mean of mean reliability score for each mouse of all neurons active on the stem across all session-pairs. Box plots show median and 1st/3rd quartiles, whiskers extend to 1.5x interquartile range. **c** Mean discriminability along the stem +/- 1 days from splitter onset for one representative mouse. p = 9.3x10-6 Kruskal-Wallis ANOVA, N = 233 neurons, *p = 7.6x10-6, **p = 0.016 two-sided post-hoc Tukey test. Same conventions as a. **d** Mean discriminability +/- 10 days from splitter onset for all mice. p = 0.015 Kruskal-Wallis ANOVA, N = 67 mean discriminability scores, *p=0.034 two-sided post-hoc Tukey test. Circles = mean of mean discriminability score for each mouse of all neurons active on the stem for across session-pairs. Same conventions as b.

Supplementary Figure 7

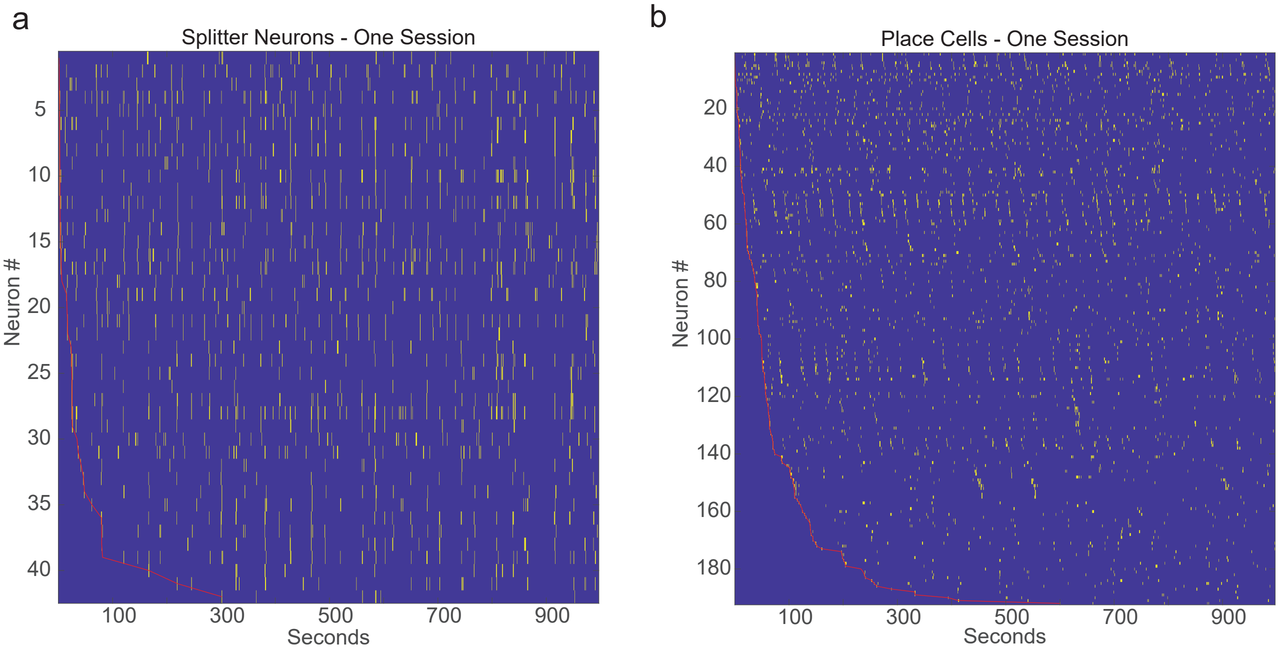

Supplementary Figure 7 | Splitter vs. Place Cell Recruitment within a Session. Related to Figure 7.

a Raster plot of calcium events (yellow) for all splitter neurons sorted by time of first calcium transient (red line). **b** Same as **a** but for place cells from the same session.
