# Supplementary material for: Trajectory-modulated hippocampal neurons persist throughout memory-guided navigation
Source: Nat Commun. 2020 May 15;11:2443. doi: 10.1038/s41467-020-16226-4 (PMC7229120; doi:10.1038/s41467-020-16226-4)
Supplement: Supplementary file 3 — Reporting Summary [file 41467_2020_16226_MOESM3_ESM.pdf]

nature research

Corresponding author(s): Nat Kinsky

Last updated by author(s): Apr 2, 2020

Reporting Summary

Nature Research wishes to improve the reproducibility of the work that we publish. This form provides structure for consistency and transparency in reporting. For further information on Nature Research policies, see Authors & Referees and the Editorial Policy Checklist.

Statistics

For all statistical analyses, confirm that the following items are present in the figure legend, table legend, main text, or Methods section.

n/a	Confirmed	
☐	☒	The exact sample size (n) for each experimental group/condition, given as a discrete number and unit of measurement
☒	☐	A statement on whether measurements were taken from distinct samples or whether the same sample was measured repeatedly
☐	☒	The statistical test(s) used AND whether they are one- or two-sided<br>*Only common tests should be described solely by name; describe more complex techniques in the Methods section.*
☐	☒	A description of all covariates tested
☐	☒	A description of any assumptions or corrections, such as tests of normality and adjustment for multiple comparisons
☐	☒	A full description of the statistical parameters including central tendency (e.g. means) or other basic estimates (e.g. regression coefficient) AND variation (e.g. standard deviation) or associated estimates of uncertainty (e.g. confidence intervals)
☐	☒	For null hypothesis testing, the test statistic (e.g. F, t, r) with confidence intervals, effect sizes, degrees of freedom and P value noted<br>*Give P values as exact values whenever suitable.*
☒	☐	For Bayesian analysis, information on the choice of priors and Markov chain Monte Carlo settings
☒	☐	For hierarchical and complex designs, identification of the appropriate level for tests and full reporting of outcomes
☐	☒	Estimates of effect sizes (e.g. Cohen's d, Pearson's r), indicating how they were calculated

Our web collection on statistics for biologists contains articles on many of the points above.

Software and code

Policy information about availability of computer code

Data collection	Data were collected using miniature fluorescence miscroscopes from Inscopix (v1/v2) and nVistaHD software (Inscopix, v2/v3).
Data analysis	Motion correction and downsampling of imaging data was performed using Mosaic v2/v3 (Inscopix) software. All custom-written MATLAB (2019a) code used in this study is freely-available at https://github.com/SharpWave/Tenaspis (neuron and calcium event extraction from raw imaging movies) and https://github.com/nkinsky/ImageCamp (all other data analysis).

For manuscripts utilizing custom algorithms or software that are central to the research but not yet described in published literature, software must be made available to editors/reviewers. We strongly encourage code deposition in a community repository (e.g. GitHub). See the Nature Research guidelines for submitting code & software for further information.

Data

Policy information about availability of data

All manuscripts must include a data availability statement. This statement should provide the following information, where applicable:

- Accession codes, unique identifiers, or web links for publicly available datasets
- A list of figures that have associated raw data
- A description of any restrictions on data availability

We will upload all raw data used in this study to a public repository and provide accession codes/links prior to publication. In the interim, we have added the following statement to the manuscript: "The data used in this study are available from the corresponding author upon reasonable request."

Field-specific reporting

Please select the one below that is the best fit for your research. If you are not sure, read the appropriate sections before making your selection.

☒ Life sciences ☐ Behavioural & social sciences ☐ Ecological, evolutionary & environmental sciences

For a reference copy of the document with all sections, see nature.com/documents/nr-reporting-summary-flat.pdf

Life sciences study design

All studies must disclose on these points even when the disclosure is negative.

Sample size	Five mice were used in this study - one was excluded after all recordings were finished (see data exclusions below). This number is based on the number of animals used/needed in previous, similar studies to obtain significant results.
Data exclusions	One mouse was excluded from this study due to an inability to correct motion-artifacts in his imaging data. 3 of 10 sessions from another mouse were excluded due to poor imaging quality/improper imaging plane. In order to exclude spurious identification of splitters we applied the following pre-established criteria: we only included sessions where mice performed at least 20 free trials and excluded neurons that produced a calcium event on the stem of the maze on fewer than 5 trials. Several between-session registrations (all at >2 weeks apart) were excluded for two mice due to significant drift of the imaging plane between sessions. In addition, based on reviewer input we excluded a number of neurons exhibiting long calcium kinetics (~13% of all neurons). This is noted clearly in the text and methods section. The decision to exclude data due to poor imaging quality and long calcium kinetics was not pre-established.
Replication	We utilized multiple analysis techniques to verify the robustness of our results and/or varied parameters used in each analysis to ensure that any observed results were not due to an arbitrary choice of parameter. Specifically, we cross-validated our method for identifying splitter neurons with previously published method (Wood et al., 2000). We found that ~90% of the neurons we classified as splitters also passed the criteria used by Wood et al. (2000), successfully replicating their results. The neurons not meeting both criteria were not classified as splitter neurons. When calculating correlations between trajectory-dependent activity and memory performance, multiple metrics were used to quantify the level of trajectory-dependent activity present in the neuronal population and verify the robustness of the result (Figure 3 and Supplementary Figure 3). 8 of 12 metrics shown in Figure 3 and Supplementary Figure 3 produced correlations that were significant or approached significance. To ensure that our finding that the higher likelihood splitter neurons remain active is not explained by higher activity rates of splitter neurons (Figure 5) we repeated the analysis removing non-splitter neurons with low event rates (Supplementary Figure 5). Our finding that removing low event-rate neurons increases the likelihood non-splitter neurons remain active indicates that both event-rate and functional coding properties of a neuron influence its subsequent activity levels.
Randomization	Only one experimental group was utilized in this study.
Blinding	No blinding was performed in this study because there was only one experimental group and all behavioral analyses were automated. All individual and group statistics utilized within animal comparisons between different functional neuron types.

Reporting for specific materials, systems and methods

We require information from authors about some types of materials, experimental systems and methods used in many studies. Here, indicate whether each material, system or method listed is relevant to your study. If you are not sure if a list item applies to your research, read the appropriate section before selecting a response.

Materials & experimental systems

n/a	Involved in the study	
☒	☐	Antibodies
☒	☐	Eukaryotic cell lines
☒	☐	Palaeontology
☐	☒	Animals and other organisms
☒	☐	Human research participants
☒	☐	Clinical data

Methods

n/a	Involved in the study	
☒	☐	ChIP-seq
☒	☐	Flow cytometry
☒	☐	MRI-based neuroimaging

Animals and other organisms

Policy information about studies involving animals; ARRIVE guidelines recommended for reporting animal research

Laboratory animals	Wild type C57/BL6 male mice aged 3-14 months were used in this study. The Boston University Animal Care and Use Committee mandates housing conditions of 18-25°C and 30-70% humidity
Wild animals	No wild animals were used in this study.
Field-collected samples	No field-collected samples were used in this study.

Ethics oversight	All procedures were performed in compliance with the Boston University Animal Care and Use Committee.

Note that full information on the approval of the study protocol must also be provided in the manuscript.
